# The Dissipation of Cyazofamid and Its Main Metabolite CCIM during Wine-Making Process

**DOI:** 10.3390/molecules25040777

**Published:** 2020-02-11

**Authors:** Qingxi Yang, Shiwei Wei, Na Liu, Zumin Gu

**Affiliations:** 1 Department of Pesticide Science, Plant Protection College, Shenyang Agricultural University, Shenyang 110866, China; yqx17635426236@163.com (Q.Y.); xrsqmhyjxyh@163.com (S.W.); 2 State Key Laboratory of Animal Nutrition, Institute of Animal Science, Chinese Academy of Agricultural Sciences, Beijing 100193, China; liuna88@126.com

**Keywords:** cyazofamid, CCIM, grape wine, procession

## Abstract

Few studies have focused on the residues of cyazofamid and its main metabolite CCIM (4-chloro-5-p-tolylimidazole-2-carbonitrile) in the wine making process, which is crucial to evaluate the potential food risk of cyazofamid and CCIM. In this work, detailed study has been conducted on the evaluation of the fate of cyazofamid and its main metabolite CCIM during the wine-making process. The targeted compounds cyazofamid and CCIM were separated and determined by high-performance liquid chromatography coupled with tandem mass spectrometry (HPLC-MS/MS) and processing procedure including washing, peeling, fermentation, and clarification. Results showed that residues of cyazofamid and CCIM decreased significantly in wine processing. The dissipation of cyazofamid in the fermentation process followed the first-order of kinetics, and the half-life of cyazofamid was 46.2–63.0 h, whereas, the residues of CCIM, in the three treatments, decreased with time elapse. The processing factors (PFs) were all less than one in different processing processes, and the PFs ranges of cyazofamid and CCIM were 0.003–0.025 and 0.039–0.067 in three treatments in the overall process. The outcome indicated that the whole process could significantly reduce the residues of cyazofamid and CCIM in red and white wines. The results might provide more precise risk assessments of cyazofamid in the wine-making process.

## 1. Introduction

**Figure 1:**
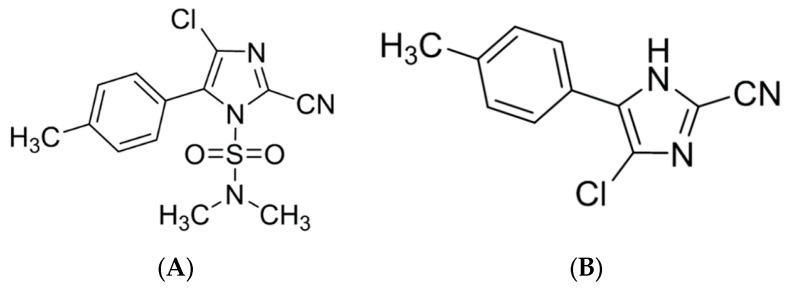
The chemical structures of cyazofamid and CCIM. (**A**) Cyazofamid; (**B**) CCIM.

Grape is widely planted worldwide, and has grown to be an indispensable part of the modern diet [1]. While grapes are consumed as a fresh fruit and also as processed products including grape juice, wine, raisins, jam, and so on, wine is the most important processed product [2,3]. At present, the most consumed wines on the market are red wines and white wines, where white wine is mainly made from white grapes or peeled grapes [4,5,6]. Research has shown that wine can effectively reduce the risk of cardiovascular disease and oxidative damage, which makes its popularity among consumers [7,8,9]. However, the occurrence of disease in vineyards, especially fungal diseases, is one of the most significant economic losses [10]. Common fungal diseases of cultivated and wild grapes in world are grey mold (*Botrytis cinerea*), powdery mildew (*Uncicula necator*), and downy mildew (*Plasmopara viti-cola*) [11]. Cyazofamid, as showed in Figure 1A, presents strong activity against Plasmodium and Oomycetes and has been widely used to control a variety of fruit and vegetable diseases such as grape downy mildew (*Plasmopara viti-cola*) [12,13]. The mode of action of cyazofamid is to interfere with the supply of energy by blocking the electron transfer at the Qi (ubiquinone reducing site) center of complex III of the enzyme cytochrome bc1 complex in the mitochondrial respiratory chain, thereby affecting the synthesis of ATP [14,15]. However, previous studies have already indicated that cyazofamid has an adverse effect on cortical neuronal cells, resulting in a significant decrease in survival rate, and it was found through subchronic toxicity tests that cyazofamid had high renal toxicity in male rats [16,17]. Moreover, after the application of cyazofamid in the field, it will rapidly decompose into CCIM in plants, as shown in Figure 1B. Studies have shown that CCIM is more easily absorbed than cyazofamid and is more toxic for rats [18]. Excessive levels of CCIM residues in agricultural products may lead to higher dietary risk [18,19]. Jin and Čuš pointed out that some pesticide residues in grapes and wine exceed the standard [20,21]. Additionally, excessive pesticide residues in wine will affect the quality of wine and threaten human health. Food security has been gradually taken seriously by various countries [22,23]. Furthermore, the issue of pesticide residues caused by food contamination has attracted more and more attention from consumers [24]. Thus, it is of great significance to monitor the fate of cyazofamid and CCIM during wine fermentation, which will be conducive to the improvement of wine-making technology and the protection of human dietary safety.

The nutritional value and flavor of most agricultural products will be improved after commercial processing [25]. In recent years, there have been many reports about the impact of processing on pesticide residues in agricultural products [26]. Different food processing technologies have distinct impacts on pesticide residues in products. Some of them may significantly reduce pesticide residues in products such as peeling, while others may increase pesticide residues such as drying [27,28,29,30]. So far, numerous researchers have studied the changes of pesticide residues in wine processing [31,32,33]. To our knowledge, many studies have reported on the residues of cyazofamid and its metabolite, CCIM, in the field [10,11,34], but little attention has been paid to the change in their residue levels caused by the process of wine-making. In order to guarantee food safety for consumers, we utilized grapes harvested in the field to carry out wine-making processing experiments. The processing factor (PF: the ratio of residue level in the processed products and that in the respective raw products) are indispensable when assessing the risk associated with the intake of pesticide residues [35]. Hence, in the process of wine-making, it is of great significance to clarify the processing factors of cyazofamid and CCIM. 

The objectives of the present study were to: (1) investigated the dissipation of cyazofamid and formation of its main metabolite CCIM during red wine and white wine fermentation; (2) provide information of cyazofamid and CCIM regarding PFs in wine-making processing including washing, peeling, fermentation, and clarification. The results of this study may provide more accurate information for evaluating the wine safety induced by cyazofamid.

## 2. Results and Discussion

### 2.1. Method Validation

Linearity was evaluated by preparing four different calibration curves (solvent, grape, pomace, and wine) with concentrations ranges from 5 to 5000 µg/L for each compound. Satisfactory linearity was observed with the correlation coefficient (R^2^) ranging from 0.9981 to 0.9997. Mean recoveries and RSDs of cyazofamid and CCIM were measured by spiking the blank samples (raw grapes, pomace, and wine) at various concentration levels (5, 100, 1000, and 5000 ug/kg) and performing quintuplicate analysis (Table 1). The recoveries were calculated by the analysis of the target compounds in the three matrices. As shown in Table 1, the mean recoveries of cyazofamid ranged from 83% to 113% with 0.4–6.6% intra-day RSDs, whereas they were 80–98% with 0.3–6.4% intra-day RSDs for CCIM. Customarily, the RSD range of the proposed method intra-day (*n* = 5) and inter-day (*n* = 15) were 0.3% to 6.6% and 0.9% to 8.8%, respectively. As indicated in Table 1, the mean recoveries of pomaces were significantly lower than that of other matrices, which may be related to the higher octanol/water partition coefficient of cyazofamid, so that the solid adsorption capacity of cyazofamid was higher. The limit of quantification (LOQs) for cyazofamid and CCIM were evaluated at the lowest spiked concentration. In this study, the LOQs of cyazofamid and CCIM in three different matrices were both 5 µg/kg.

### 2.2. Effects of Processing

The corresponding concentration of cyazofamid and CCIM in the processed commodity is shown in Table 2. Usually, the first step in most processing procedures is washing, which is a necessary step to remove pesticide residues in raw fruit, and many studies have comprehensively and thoroughly revealed the influence of the washing process to remove pesticide residues in agricultural products [30,36,37,38]. In this work, the raw grapes were washed with running water for 10 min. As presented in Table 2, after the washing process, the mean loss of cyazofamid and CCIM were 37.0% and 31.0%, respectively, which was consistent with the results obtained by Han et al. [39]. Han found that the mean loss of spirotetramat and spirotetramat-enol by washing was significant, with a 40.6% loss of spirotetramat and 32.2% loss of spirotetramat-enol [39]. Meanwhile, Liu [36] and Han [38] found that washing had less impact on the reduction of tetraconazole and pyridaben, respectively. The removal rate of tetraconazole in strawberries by Liu was 25%, and that of pyridaben in apples by Han was 5.7%. The octanol/water partition coefficient of cyazofamid, tetraconazole. and pyridaben were 3.20, 3.56, and 6.37, respectively [40]. The results indicated that the removal rate of pesticides by washing may be related to the octanol/water partition coefficient. The larger the octanol/water partition coefficient, the smaller the solubility of the compound in water, and the less obvious the removal effect by washing.

The grapes were peeled after washing. Peeling is, as indicated in Table 2, another important step in the processing procedure of winemaking. The data indicated that the concentrations of cyazofamid and CCIM in the unwashed grape skin were the highest, and showed that peeling had a notable effect on the reduction of cyazofamid and CCIM with a 95.0% and 78.0% decrease, respectively. Previous studies have found that peeling could eliminate most pesticides in agricultural products [41,42]. By comparing the residual amount of cyazofamid in unwashed grape skins and peeled grapes, it was found that the residual amount of the latter was much lower than the former. The results showed that wax of the cuticular may play a critical character in preventing the deposition of pesticides in the grape fruit [43]. Furthermore, the results also fully demonstrated that the removal of cyazofamid and CCIM residues was more effective than washing. This was because the washing step only reduced the pesticide residues that were loosely attached to the surface of the fruit, and peeling could even remove the pesticides that had penetrated into the grape skin [44]. 

Then, the next step was the fermentation process, which began with the crushing of the grapes. In this work, unpeeled (Groups A, B) and peeled (Group C) grapes were used as raw materials to study the wine-making. After the crushing process, the pesticide enters a two-phase system consisting of a liquid phase and a solid phase, which is distributed between the two phases [45]. As shown in Table 2, the mean loss of cyazofamid and CCIM were both 95.4% and 88.0% in Group A and 92.6% and 91.1% in Group B, respectively, after fermentation. The residual amount of CCIM in wine after fermentation in Group C was less than the limit of quantification (LOQ = 5 ug/kg), and the mean loss of cyazofamid was 89.5%. In comparison, pesticide residues in the byproducts (pomace) was significantly higher than that of wine. The results showed that cyazofamid was mostly retained in the solid phase, which may be related to the higher octanol/water partition coefficient of cyazofamid, which made the adsorption capacity of solid to cyazofamid relatively higher. The byproduct of wine making can be used to produce grappa, so the study on the residue of cyazofamid in the byproduct cannot be ignored [45]. Finally, bentonite was added for clarification. Bentonite was the most commonly used fining agent in the process of wine making and it can absorb the suspended proteins, metal ions, and yeast cells in the wine to make the wine clear [46]. As indicated in Table 2, the mean loss of cyazofamid and CCIM were both 36.1% and 33.9% in Group A and 38.6% and 24.5% in Group B, respectively, after clarification. In Group C, the residual amount of CCIM in wine after clarification was less than the limit of quantification (LOQ = 5 ug/kg), and the mean loss of cyazofamid was 40.1%. This may be mainly because bentonite has a strong adsorption capacity for cyazofamid and CCIM. Concentrations of cyazofamid and its metabolite CCIM in the final grape wine were also high (Table 2), which reminded us that we should put the detection of wine dietary safety in an important position.

### 2.3. Degradation of Cyazofamid and Its Metabolite CCIM During Wine-Making Process

**Figure 2:**
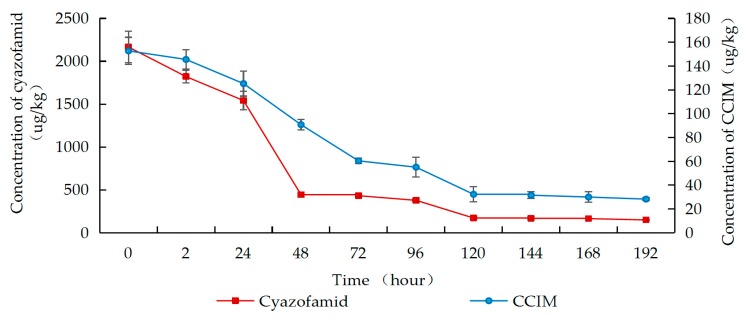
Concentrations of cyazofamid and CCIM in Group A during the wine-making process.

**Figure 3:**
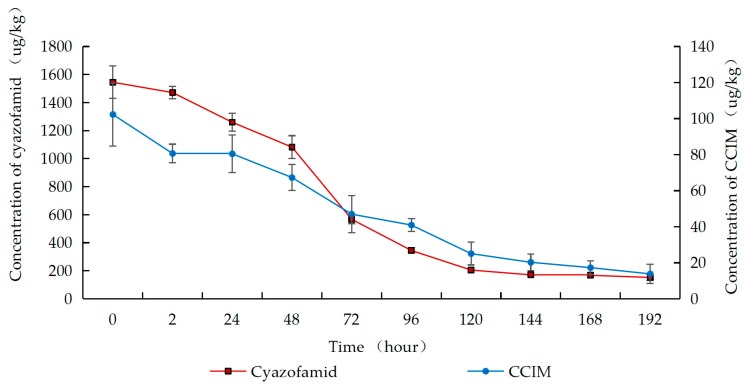
Concentrations of cyazofamid and CCIM in Group B during the wine-making process.

**Figure 4:**
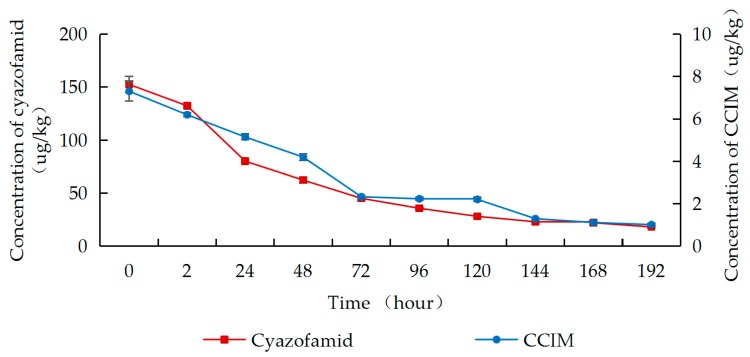
Concentrations of cyazofamid and CCIM in Group C during the wine-making process.

As shown in Figure 2, Figure 3 and Figure 4, residues of cyazofamid and CCIM decreased with the increase in fermentation time. In Groups A–C, the dissipation of cyazofamid in the fermentation process followed the first-order of kinetics (R^2^ = 0.8920 in Group A, R^2^ = 0.9478 in Group B, and R^2^ = 0.9495 in Group C). The half-life of cyazofamid was 46.2 h in Group A, 49.5 h in Group B, and 63.0 h in Group C, respectively. As seen in Figure 2, Figure 3 and Figure 4, in the three treatments, the residues of CCIM at the start of fermentation (0 h) were 152.8, 102.3, and 7.3 ug/kg, respectively, which gradually decreased with time elapse. In Group C, after 24 h of fermentation, the residual amount of CCIM was less than the limit of quantification. Comparing the half-life of the three groups, the half-life of cyazofamid in Group C was longer than that in Groups A and B, which may be due to the small number of yeast in Group C. A large number of yeasts were attached to the grape skin, while in the early stage of fermentation, the grape skin of Groups A and B would float on the top, reducing heat dissipation. The higher temperature in the fermentation tank was more conducive to the reproduction of yeast, making the amount of yeast in Groups A and B more than that in Group C. Then, the high yeast amount may accelerate the dissipation of cyazofamid. The results of this work were similar to previous reports that yeast had the capability to degrade some pesticides and reduced the residues in wine [45]. The results can provide a basis for the risk assessment of cyazofamid in wine-making process. Based on previous research, CCIM was formed in the first step in the degradation of cyazofamid, which was then converted to 4-(4-chloro-2-cyanoimidazole-5-yl) benzoic acid (CCBA) or 4-chloro-5-p-tolylimidazole-2-carboxylic acid (CTCA) through different pathways [47,48], and CCIM and CTCA have been shown to be more acutely toxic than cyazofamid [19].

### 2.4. Processing Factors

The Joint Meeting of Pesticide Residues (JMPR) stipulates that the purpose of residue research in food processing is to correlate the residue content of processed commodities with the residue content of agricultural raw materials and to calculate processing factors (PFs) experimentally. The formula for calculating PF is as follows:

(1) $$\mathrm{PFs}=\frac{\mathrm{residue}\;\mathrm{level}\;\mathrm{in}\;\mathrm{processed}\;\mathrm{commodity}}{\mathrm{residue}\;\mathrm{level}\;\mathrm{in}\;\mathrm{the}\;\mathrm{raw}\;\mathrm{agricultural}\;\mathrm{commodity}}$$

Equation (3) showed that when the PF value is less than 1, the pesticide residues in the processing of agricultural products is reduced. Conversely, when the PF value is greater than one, the pesticide residue is increased [27]. The PFs of cyazofamid and CCIM during the wine-making process were evaluated and presented in Table 3. The results showed that the PFs were all less than one in different processing processes, indicating that each process had the effect of reducing the residues of cyazofamid and CCIM. The results showed that the overall process PFs ranges of cyazofamid and CCIM were 0.003–0.025 and 0.039–0.067 in the three treatments, respectively, indicating that the whole process could significantly reduce the residues of cyazofamid and its metabolites in red and white wine. The results were similar to a previous report, where the wine-making process (washing, crushing, fermentation, clarification) could reduce the concentration of pesticide residues to a certain extent [49].

## 3. Materials and Methods 

### 3.1. Materials

The analytical standard cyazofamid (98.9% purity) and its metabolite CCIM (96.18% purity) were obtained from Dr Ehrenstorfer (Augsburg, Germany). Commercial 20% cyazofamid suspension concentrate (SC) was obtained from Zhejiang Tianfeng Biological Science Co., Ltd. (Zhejiang, China). HPLC-grade methanol and acetonitrile were purchased from Fisher Scientific (Shanghai, China). Analytical grade acetonitrile, anhydrous magnesium sulfate, and sodium chloride for pesticide residue analysis were purchased from Beijing Chemical and Reagent (Beijing, China). Graphitized carbon black (GCB, 40 μm) and primary secondary amine (PSA, 40 μm) were obtained from Agela Technologies (Tianjin, China). Ultrapure water was prepared by a Milli-Q reagent water system (Millipore, Bedford, MA, USA). 

The standard stock solution (100 mg/L) of cyazofamid and CCIM was prepared in HPLC-grade acetonitrile. The working solution and calibration were prepared by the appropriate dilution of the stock solution at the concentrations of 5, 50, 100, 500, 1000, and 5000 ug/L on the day of analysis. Correspondingly, the matrix-matched standard solution was prepared at the same concentration by adding blank grape, wine, and pomace sample extracts to each serially diluted standard solution, respectively. All the solutions were stored at 4 °C in the dark.

### 3.2. Field Experiments

The field trials were conducted in a commercial orchard located in Liaoning Province, China, which were surveyed and determined free of cyazofamid and CCIM before the experiment. Three replicates and one blank control were established, with a total of four experimental plots, each with an area of 30 m^2^. Based on the Organization for Economic Co-Operation and Development OECD guidelines for pesticide residues in processed commodities [50], during grape maturation, the cyazofamid commercial product (20% SC) was applied on grape with the foliar spraying mode at a triple higher dosage of the commercial recommendation of 200.1 g active ingredient per hectare. The recommendation dosage is 66.7 g active ingredients per hectare. The commercial product (20% SC) was sprayed three times with a LP-605 manual sprayer (Agrolex, Singapore) on 15, 22, and 29 August 2018. Approximately 50 kg of grape samples in the mature stage were harvested three days after the last pesticide application. All of the above samples were transported to the laboratory and processed immediately.

### 3.3. Winemaking and Sampling

**Figure 5:**
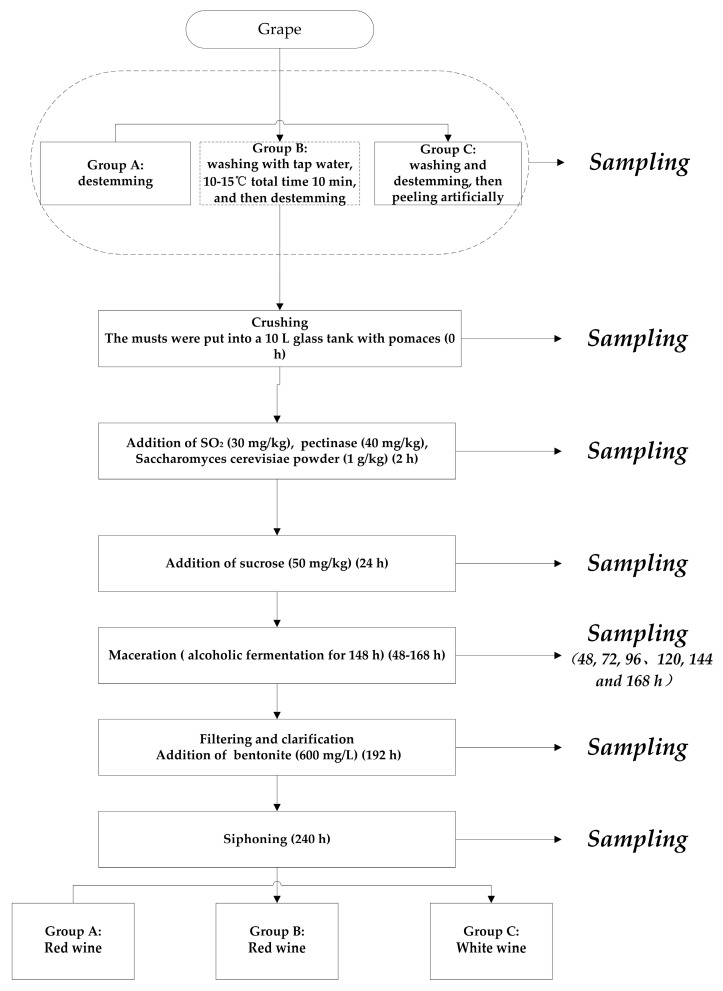
Scheme for wine-making process used in this study and the sampling points.

For each treatment, winemaking trials were carried out using the technology currently used in wineries, similar to the methods outlined by Pan et al., Grazioli et al., and Leong et al. [4,5,6]. The winemaking experiment was divided into three groups (Groups A, B, C) as shown in Figure 5. Each treatment weighed about 5 kg of grapes to be treated differently before the crushing procedure: Group A: destemming; Group B: washing with tap water, 10–15 °C total time 10 min, and then destemming; and Group C: washing and destemming, then peeling artificially. The same procedures were then performed in three groups. The first step was to crush the grapes and the musts were put into a 10 L glass tank with pomaces containing the skins and seeds. Then, 30 mg/kg of SO_2_, 40 mg/kg of pectinase, and 1 g/kg of Saccharomyces cerevisiae powder were added in turn. After 24 h, 50 g/kg of sucrose was approximately added to the must. Place the fermentation tank at a fermentation environment of 25 ± 1 °C for maceration and alcoholic fermentation. The must was stirred three times a day for the first three days of fermentation to ensure the maceration effect. After seven days, the alcohol fermentation was completed, and the liquid phase was separated from the pomaces by the filtration device. Then, 600 mg/L bentonite was added to the separated liquid phase for clarification and the clear wine was siphoned after two days of clarification. Ultimately, after a series of processing procedures, red wine was obtained from Groups A and B, while white wine was obtained from Group C. From field sampling to the end of clarification (240 h), each group was set with three repetitions (Figure 5). All samples were stored at −20 °C for analysis.

In this study, samples including raw grape, washed grape, peeled grape, grape skin, fermentation wine, pomace, and clarification wine in diverse processing steps were collected to determine and research the transforms and dissipation trend of the pesticide residues of cyazofamid and CCIM during the processing procedure.

### 3.4. Extraction and Clean-Up Procedure

A portion of 10.0 g homogenized samples (grape, pomaces, or wine) were weighed into a 50 mL PTFE centrifuge tube. Then, 10 mL of acetonitrile was added, and the mixture was vortexed on a Geno/Grinder mechanical shaker (SPEX Sample Prep, USA) for 5 min at 1200 strokes per min. Afterward, 1 g NaCl and 4 g anhydrous MgSO4 were added, and the shaking step was again conducted for 3 min. Then, the tubes were centrifuged for 5 min at relative centrifugal force (RCF) 2077× *g* with a TG16-WS centrifuge (Xiangyi Centrifuge Machines, China). Next, 1.5 mL of the upper layer was transferred into a single-use centrifuge tube containing the sorbent (150 mg anhydrous MgSO4 + 10 mg GCB + 50 mg PSA), then vortexed for 1 min and centrifuged for 5 min at RCF 2400× *g*. Finally, the upper layer was filtered with 0.22 μm nylon syringe filters (15 mm diameter, Agela Technologies, China) for detection.

### 3.5. Instrumentation and HPLC-MS/MS Analytical Conditions

Chromatographic separation was carried out on an Agilent 1290 high-performance liquid chromatography (HPLC) system (Milford, MA, USA) equipped with an Eclipse Plus C18 column (2.1 mm × 50 mm, 1.8 μm particle size). Gradient UPLC elution was performed with 0.2% (*v*/*v*) formic acid in water as mobile phase A and acetonitrile (chromatography grade) as mobile phase B. The elution was performed as follows: 0–0.5 min, 40% A; 1.5 min, 30% A; 2.5 min, 10% A; 3.0 min, 20% A; and 5.0 min, 40% A. The flow rate was 0.3 mL/min, and the injection volume was 1 μL. Separation of the compounds was completed within 2.0 min. The temperatures of the column and sample manerger were set 45 °C and 5 °C.

Detection was achieved using a triple-quadrupole mass spectrometer (QQQ, Agilent Technologies) equipped with the positive electrospray ionization (ESI+) mode to quantify the target compounds. The data were collected and analyzed by MassHunter Workstation Software Version B.08.00 (Agilent Technologies, CA, USA). The acquisition parameters were as follows: The capillary voltage and nozzle voltage were 4.5 kV and 500 V, respectively. The nebulizer gas and collision gas were 99.95% and 99.999% nitrogen, respectively. The sheath gas and gas temperature were 350 °C and 325 °C with the flow rate 10 and 8 L/min, respectively. 325 (*m*/*z*) was selected as the precursor ion for cyazofamid, and its quantitative and qualitative product ions were 108 (*m*/*z*) and 44 (*m*/*z*), respectively, when the fragment voltage were both 135 V, with the corresponding collision energy of 10 and 30 eV. As for CCIM, 217.9 (*m*/*z*) was selected as the precursor ion, and its quantitative and qualitative product ions were 183 (*m*/*z*) and 139 (*m*/*z*), respectively, when the fragment voltage was both 75 V, with the corresponding collision energy of 20 and 25 eV. According to the instrument conditions and our previous report [30], the elution sequence of cyazofamid and its metabolites were CCIM (1.00 min) and cyazofamid (1.62 min), respectively.

### 3.6. Recovery Assay

Recovery assays were carried out to investigate the method accuracy and precision. Five replicates of spiked samples (raw grape, pomace and wine) at different levels (5, 100, 1000, and 5000 µg/kg) were prepared on three different days. The precision and accuracy are reflected by the relative standard deviation (RSD) and recovery, respectively. Before the extraction step, the spiked samples were permitted to settle at room temperature for 30 min to evenly distribute the pesticide and ensure complete interaction with the sample matrix and then treated as described above. The recoveries obtained from the extracted spiked samples were compared to a matrix-matched calibration solution. The matrix calibration curve prepared by this method automatically corrected the data for analytical recovery.

### 3.7. Data Analysis

The fermentation test was created three times. The results of the concentration were expressed as independent test means (± SD). 

Using the first-order kinetic equation, the dissipation kinetic of cyazofamid from the start of crushing (0 h) to the end of clarification (192 h) was estimated during the fermentation process. The half-life of cyazofamid was calculated using the following equations:

(2) $$C=C_{0}e^{-kt}$$

(3) $$T_{1/2}=\ln2/k$$

where C_0_ and C represent the concentration of the cyazofamid at the initial time and time t. *k* is the dissipation rate constant.

## 4. Conclusions

In this work, the fate of cyazofamid and its main metabolite CCIM in grape samples during wine-making processing was carefully investigated. According to the significance analysis of differences, different processing procedures had different effects on the removal of residues of cyazofamid and CCIM. Washing and peeling process were quickest and most effective way to remove pesticide residues, because after spraying in the field, the cyazofamid SC first attached to the grape surface. Fermentation and clarification also had different effects on pesticide removal. Among them, through a significant difference analysis, after the process of clarification, cyazofamid and CCIM were not significantly reduced. At the same time, it should be noted that pesticides are mostly concentrated in the grape skin and byproducts of wine, and the grape skin is rich in anthocyanins so the byproduct of wine making can be used to produce grappa. Therefore, we should pay attention to the safety of its commercial by-products [45,51]. The results showed that the PFs were all less than one in different processing processes, and the overall process PFs ranges of cyazofamid and CCIM were 0.003–0.025 and 0.039–0.067 in the three treatments. This indicates that the whole process could significantly reduce the residues of cyazofamid and its metabolites in red and white wine. In addition, the dissipation of cyazofamid in the fermentation process followed the first-order of kinetics, and the half-life of cyazofamid was 46.2–63.0 h. The residues of CCIM, in the three treatments, decreased with the increase of fermentation time. The results might provide more precise risk assessments of cyazofamid in the wine-making process. Meanwhile, the maximum residue limit of cyazofamid in wine has not been established internationally. The results of this study can improve the theoretical basis and data support for the formulation of residue limits in wine in the future. Previous studies have pointed out that pesticide residues can affect the wine quality during winemaking [52,53,54]. This study only studied the degradation trend of cyazofamid and its metabolites during winemaking. The effects of the volatile composition of wines were poorly understood, so further studies are needed to clarify the effect of cyazofamid on wine quality during the brewing process.

## Figures and Tables

**Table 1:** Accuracy and precision of the proposed method in grape samples at four spiked levels.

Compounds	Matrix	Spiked Level/ (ug/kg)	Intra-Day (*n* = 5)						Inter-Day (*n* = 15)
			Day 1		Day 2		Day 3		
			Mean Recoveries/%	RSDr/%	Mean Recoveries/%	RSDr/%	Mean Recoveries/%	RSDr/%	RSD_R_/%
Cyazofamid	Raw grape	5	112 ^l^	5.5	108 ^ijk^	3.1	107 ^j^	0.9	5.2
		100	108 ^kl^	3.7	103 ^hj^	2.4	105 ^ij^	1.4	3.5
		1000	98 ^gh^	4.0	100 ^gh^	5.0	101 ^ghij^	0.7	4.2
		5000	95 ^efg^	1.2	100 ^gh^	1.1	100 ^ghij^	1.6	1.3
	Wine	5	109 ^kl^	0.4	111 ^jk^	3.2	103 ^hij^	2.2	2.9
		100	109 ^kl^	2.3	103 ^hi^	0.5	104 ^ij^	0.5	3.7
		1000	100 ^hi^	4.1	100 ^gh^	6.6	94 ^defg^	4.6	4.8
		5000	99 ^ghi^	1.0	96 ^efg^	2.4	92 ^cdef^	5.2	5.6
	Pomace	5	105 ^jk^	5.2	113 ^k^	4.9	98 ^fghi^	6.3	3.9
		100	103 ^ij^	1.1	100 ^gh^	2.4	91 ^bcdef^	2.1	1.6
		1000	86 ^bc^	2.3	106 ^ij^	3.6	84 ^ab^	5.5	4.3
		5000	90 ^cd^	3.5	92 ^cde^	4.4	83 ^a^	7.8	6.0
CCIM	Raw grape	5	96 ^fgh^	1.2	95 ^efg^	0.7	89 ^abcde^	6.4	3.1
		100	93 ^def^	0.3	91 ^bcde^	2.1	91 ^bcdef^	5.9	1.9
		1000	89 ^bcd^	7.7	96 ^efg^	4.1	85 ^abc^	4.9	6.7
		5000	85 ^b^	2.3	88 ^abcd^	3.7	87 ^abcd^	6.4	3.6
	Wine	5	93 ^def^	6.2	98 ^fgh^	5.8	96 ^efgh^	3.8	2.9
		100	89 ^bcd^	5.4	87 ^abc^	3.3	94 ^defg^	0.9	8.8
		1000	91 ^de^	1.9	92 ^cde^	4.1	90 ^abcde^	1.8	6.5
		5000	93 ^def^	3.3	93 ^def^	2.2	88 ^abcd^	1.1	7.2
	Pomace	5	91 ^de^	1.7	94 ^ef^	0.9	90 ^abcde^	2.6	1.3
		100	86 ^bc^	2.6	98 ^fgh^	1.2	89 ^abcde^	1.8	2.2
		1000	89 ^bcd^	2.1	86 ^ab^	0.3	85 ^abc^	2.9	0.9
		5000	80 ^a^	2.9	85 ^a^	3.5	83 ^a^	3.1	3.1

^a^ RSDr intra-day, the relative standard deviations for repeatability (*n* = 5); RSDR inter-day, the relative standard deviations for reproducibility (*n* = 15). ^a–l^ Values with the different letters are significantly different (*p < 0.05*).

**Table 2:** The concentration of cyazofamid and CCIM of grape samples after different process. (*n* = 3).

Treatments	Compounds	Concentrations (ug/kg)						
		Raw Grape	Washed Grape	Peeled Grape	Grape Skin	Fermentation Wine	Byproduct (Pomace)	Clarification Wine
Group A	Cyazofamid	3255.1 ^b^ ± 223.6	-	-	-	149.7 ^a^ ± 3.5	3281.6 ^b^ ± 60.2	95.7 ^a^ ± 4.4
	CCIM	236.4 ^b^ ± 18.6	-	-	-	28.3 ^a^ ± 1.1	286.1 ^c^ ± 2.8	18.7 ^a^ ± 3.2
Group B	Cyazofamid	3289.3 ^c^ ± 236.9	2073.1 ^b^ ± 120.3	-	-	152.8 ^a^ ± 21.2	3267.2 ^c^ ± 151.2	93.8 ^a^ ± 15.3
	CCIM	226.4 ^d^ ± 11.0	156.2 ^b^ ± 12.3	-	-	13.9 ^a^ ± 5.3	196.9 ^c^ ± 14.2	10.5 ^a^ ± 1.3
Group C	Cyazofamid	3461.5 ^b^ ± 249.1	-	173.1 ^a^ ± 15.6	42396 ^c^ ± 500.6	18.2 ^a^ ± 0.7	203.6 ^a^ ± 11.4	10.9 ^a^ ± 1.2
	CCIM	245.6 ^a^ ± 11.2	-	54.0 ^a^ ± 5.9	3026.8 ^b^ ± 125.6	<LOQ ^1^	12.7 ^a^ ± 0.7	<LOQ ^1^

^1^ LOQ of cyazofamid and CCIM were both 5 ug/kg. ^a–d^ Values with the different letters are significantly different (*p* < 0.05).

**Table 3:** PFs for different processing procedures of cyazofamid and CCIM (*n* = 3).

Treatments	Compounds	PFs of Processing Types				
		Washing	Peeling	Fermentation	Clarification	Overall Process
Group A	Cyazofamid	-	-	0.046	0.639	0.025
	CCIM	-	-	0.120	0.661	0.067
Group B	Cyazofamid	0.630	-	0.074	0.614	0.024
	CCIM	0.690	-	0.089	0.755	0.039
Group C	Cyazofamid	-	0.025	0.105	0.599	0.003
	CCIM	-	0.187	-	-	-
